# Supplementary material for: Biomolecules of 2-Thiouracil, 4-Thiouracil and 2,4-Dithiouracil: A DFT Study of the Hydration, Molecular Docking and Effect in DNA:RNAMicrohelixes
Source: Int J Mol Sci. 2019 Jul 15;20(14):3477. doi: 10.3390/ijms20143477 (PMC6678171; doi:10.3390/ijms20143477)
Supplement: Supplementary file 1 [file ijms-20-03477-s001.pdf]

International Journal of *Molecular Sciences*

MDPI

SUPPLEMENTARY MATERIAL

Biomolecules of 2-thiouracil, 4-thiouracil and 2,4-dithiouracil: a DFT study of the hydration, Molecular Docking and effect in DNA:RNA Microhelixes

M. Alcolea Palafox*, A. Milton Franklin Benial, and V. K. Rastogi

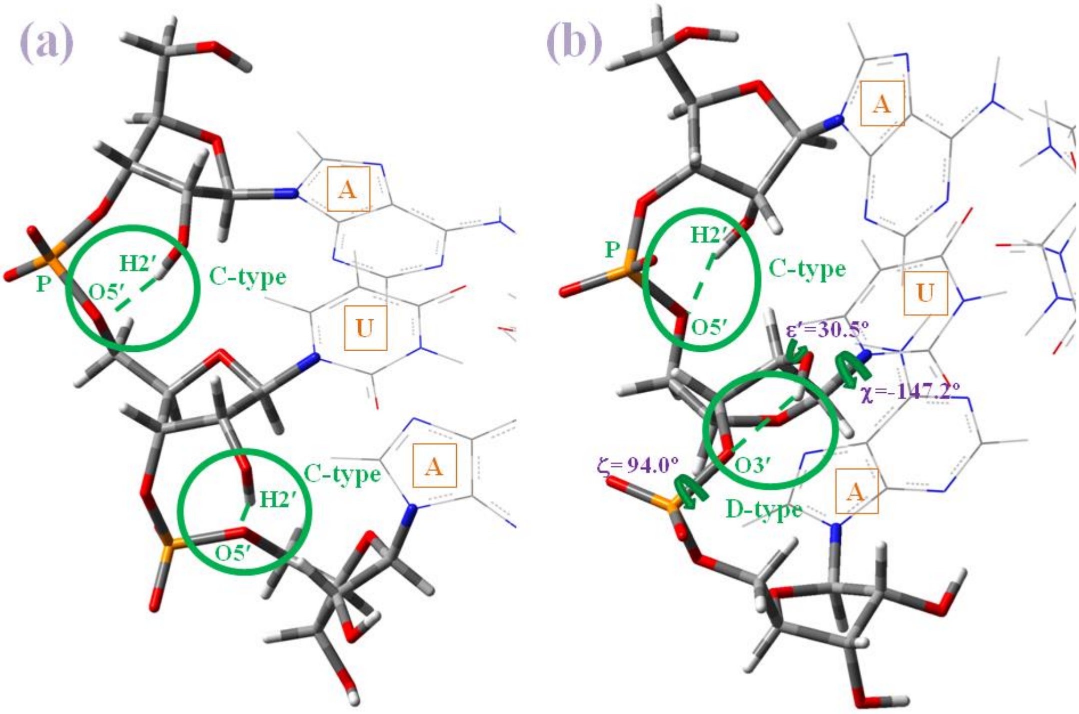

Scheme S1. Strand *I* of the 5′-AUA-3′ DNA:RNA microhelix with other two different kinds of intermolecular H-bonds: (a) C-type with intra-strand H-bonds O2′-H2′(n)···O5′(n+1). (b) With C-type and D-type in the same strand. D-type corresponds to the intra-strand H-bonds O2′-H2′(n)···O3′(n)

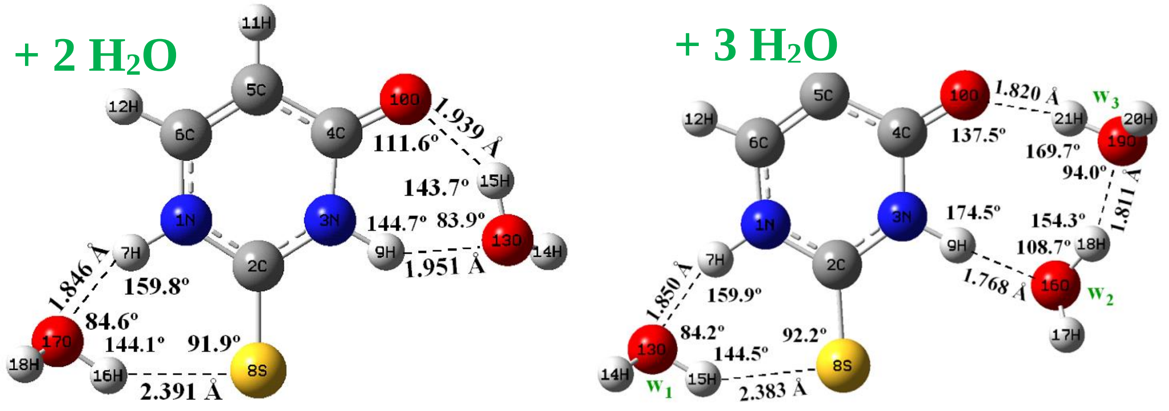

-890.724588 (-890.763584), μ = 3.638 D -967.175637 (-967.218911), μ = 4.775 D

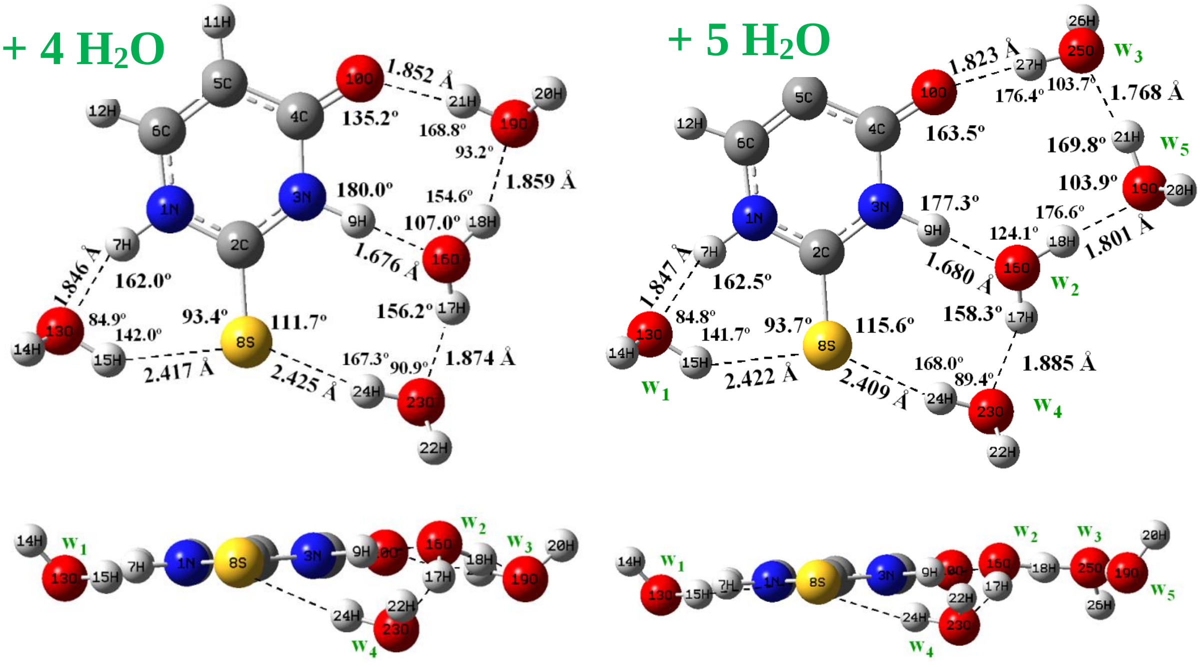

-1043.623837 (-1043.670705), μ= 5.025 D -1120.072089 (-1120.124651), μ= 5.064 D

Fig. S1. Optimized most stable hydration clusters of 2-thiouracil with two to five water molecules at the B3LYP/6-311+G(2d,p) level. Two views of each cluster are plotted. The total energy+ZPE (*E*) and the Gibbs energy (*G*) (in parentheses) are in atomic units. The dipole moment (μ) is in Debyes.

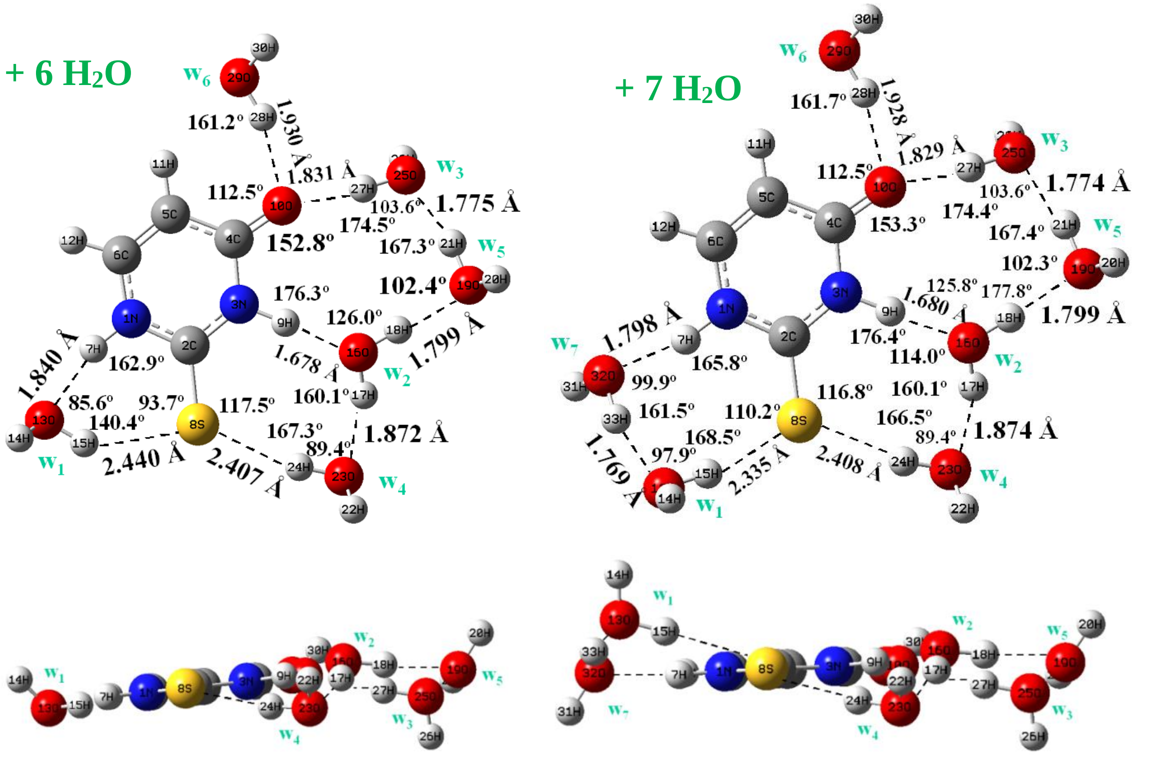

E= -1196.517128 AU (*G*= -1196.574563 AU), μ= 4.293 D *E*= -1272.967966 AU (*G*= -1273.029865 AU), μ= 2.572 D

Fig. S2. Optimized most stable hydration clusters of 2-thiouracil with six and seven water molecules at the B3LYP/6-311+G(2d,p) level. Two views of each cluster are plotted. The total energy+ZPE (*E*) and the Gibbs energy (*G*) (in parentheses) are in atomic units. The dipole moment (μ) is in Debyes.

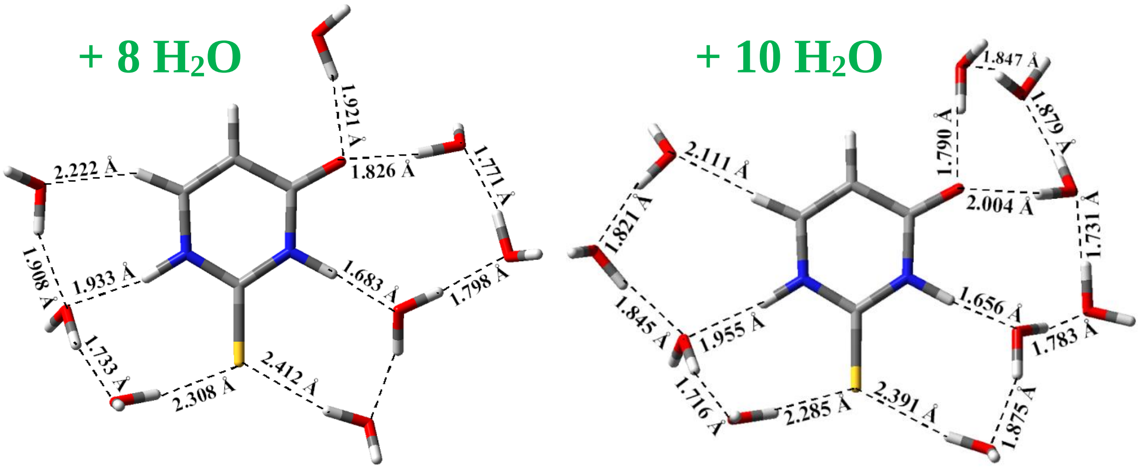

-1349.414376 (-1349.480530 AU), μ= 5.211 D -1502.313201 (-1502.385980 AU), μ= 6.788 D

Fig. S3. Optimized most stable hydration clusters at the B3LYP/6-311+G(2d,p) level of 2TU with 8 and 10 water molecules. The total energy+ZPE and the Free energy G (in parentheses) are in atomic units. The dipole moment (μ) is in Debyes.

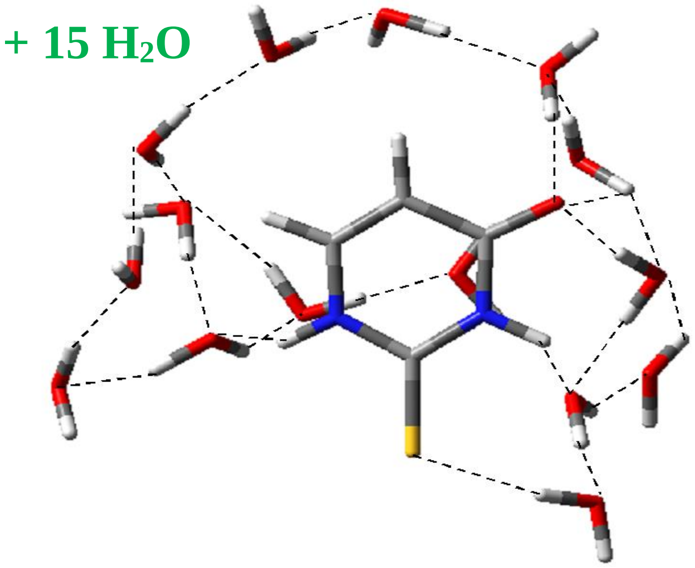

E = -1883.944172 AU (*G* = -1884.021061 AU), μ=2.289 D

Fig. S4. Optimized most stable hydration cluster with 15 water molecules at the B3LYP/6-311G(d,p) level.

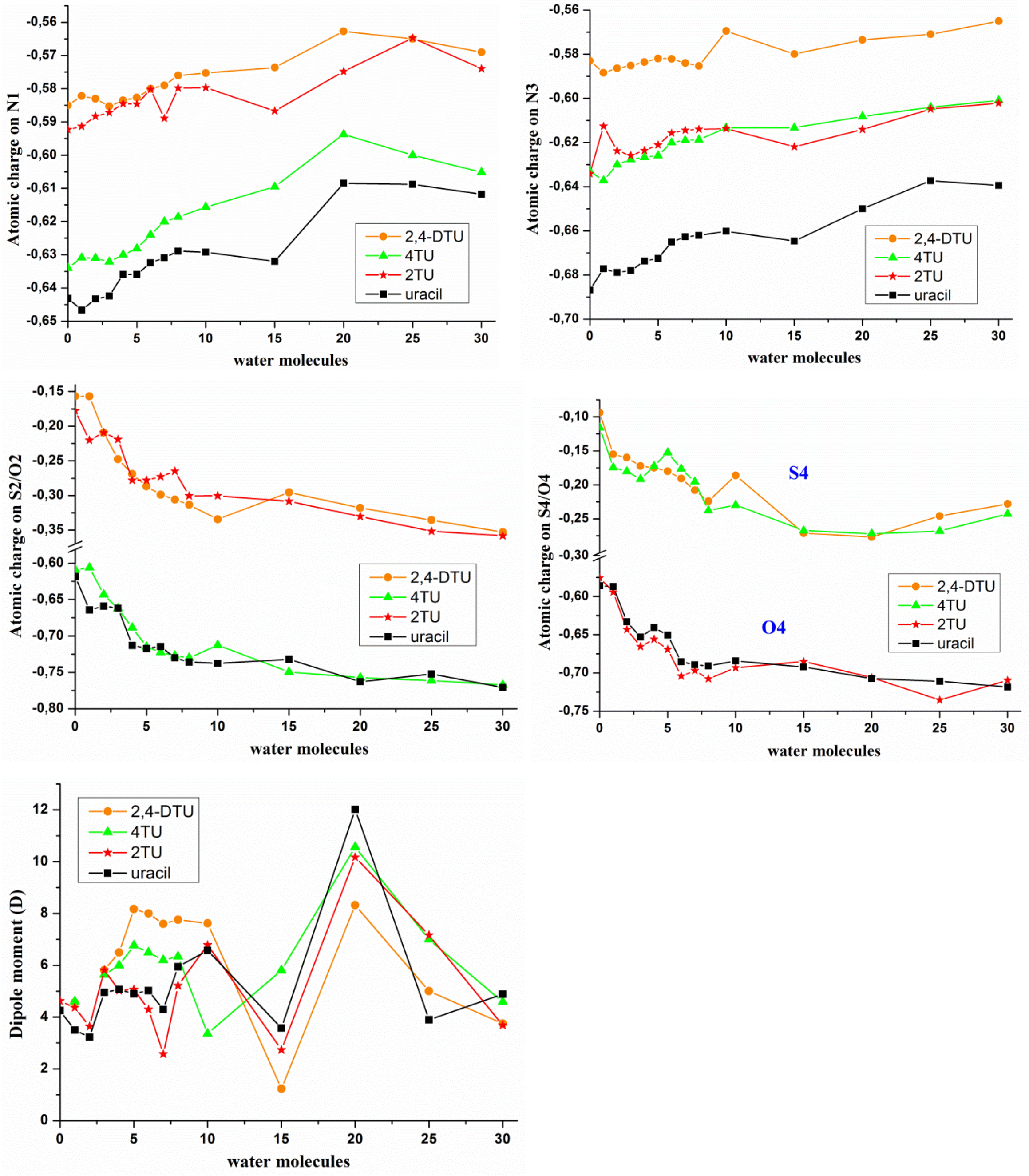

Fig. S5. Variation observed in the NBO atomic charges of the nitrogen, oxygen and sulphur atoms with the progress of the hydration up to 30 water molecules. Disparity determined in the dipole moment values with the hydration. Comparison of uracil versus 2-thiouracil, 4-thiouracil and 2,4-dithiouracil molecules. The values were calculated at the B3LYP/6–31G(d,p) level.

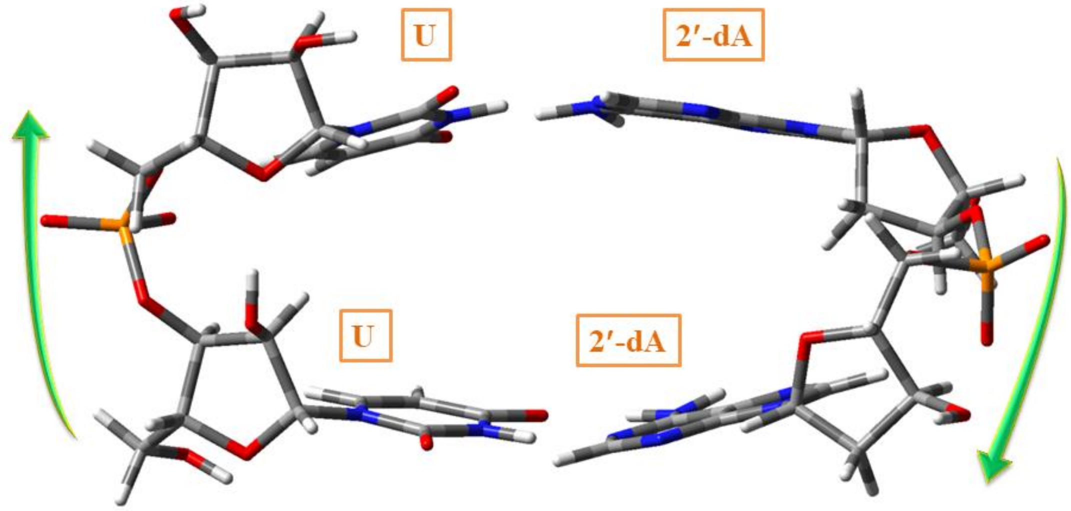

Fig. S6. Optimized microhelix with 2 nucleotide pairs at the B3LYP/6-31G(d,p) level.

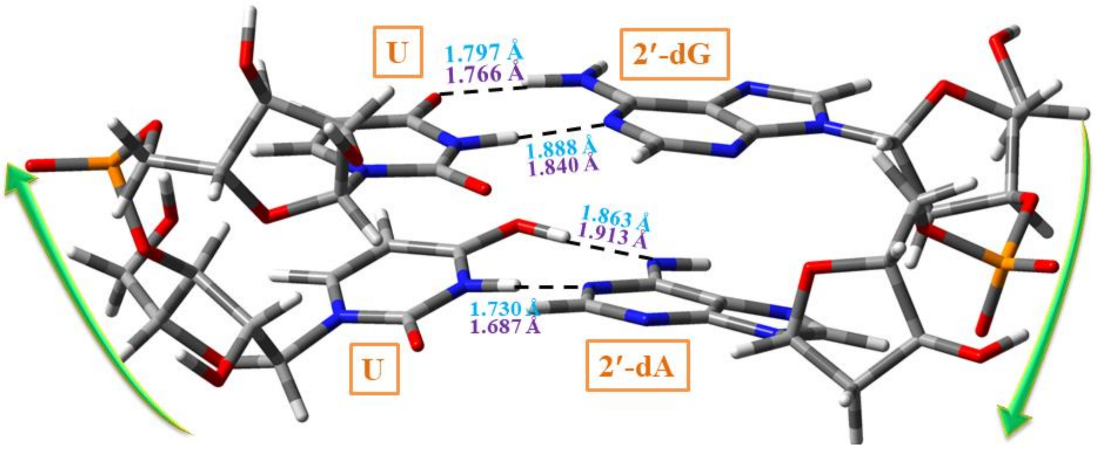

E= -4578.738027 AU (*G* = -4578.837595 AU), *E*= -4577.631312 AU (*G* = -4577.729292 AU)

Fig. S7. Intermolecular H-bonds values optimized by the DFT methods: M052X and M062X, in the microhelix with 2 nucleotide pairs corresponding to uridine: 2′-dG and uridine*: 2′-dA*. The total electronic energy (*E*) and Gibbs energy (*G*) in AU calculated with each DFT method is shown at the end of each figure.

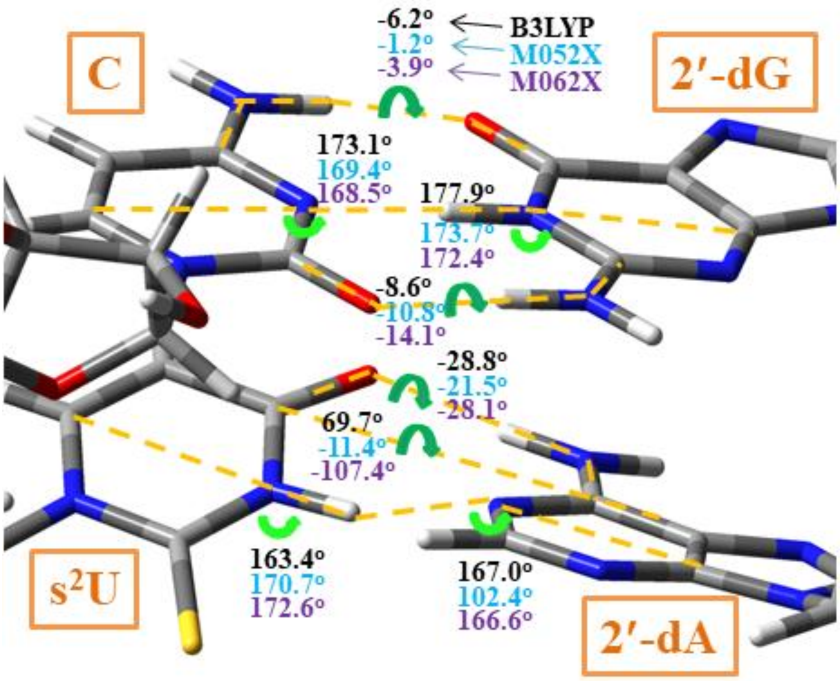

Fig. S8. Intermolecular torsional angles values optimized by the DFT methods: B3LYP, M052X and M062X, in the microhelix with 2 nucleotide pairs corresponding to cytidine: 2′-dG and 2-thiouridine: 2′-dA.

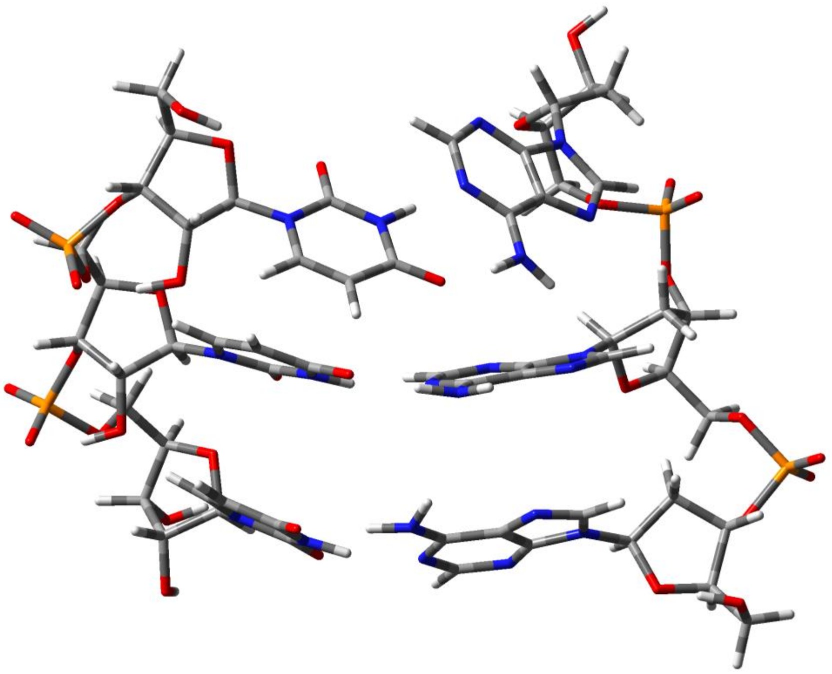

Fig. S9. Microhelix 5′-UUU-3′ with three nucleotide pairs optimized at the LC-wPBE/6-31G(d,p) level.

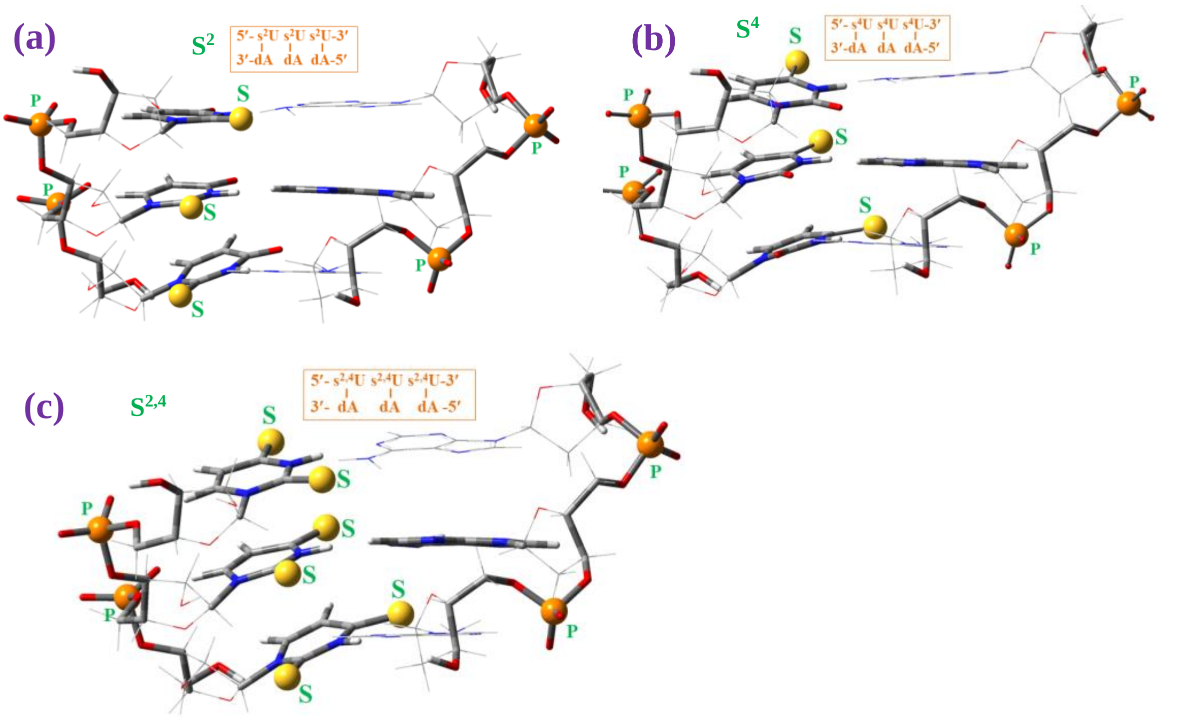

Fig. S10. Main differences observed in the deformation of the microhelixes type A with three thio-nucleotide in strand *I* of the base pairs. (a) with 2-thiouridine, (b) with 4-thiouridine, (c) with 2,4-dithiouridine.

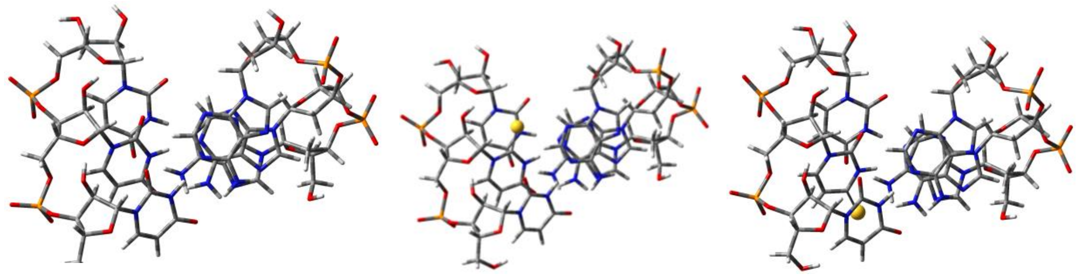

5′-UUU-3′ 5′-U s^{2}U U-3′ 5′-U s^{4}U U-3′

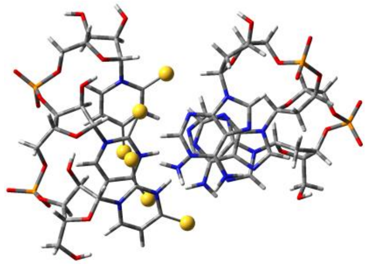

5′-U s^{2,4}U U-3′

Fig. S11. Different spacial orientation of the A-type microhelixes optimized at the M062X/6-31G(d,p) level.

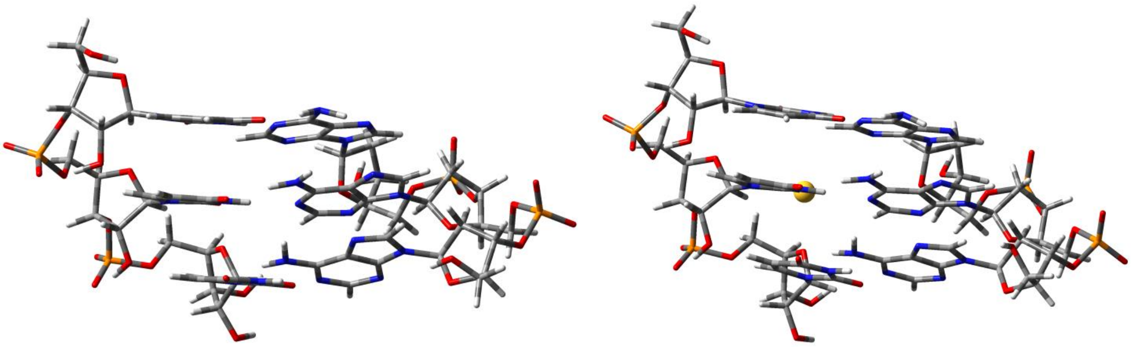

5′-UUU-3′

Fig. S12. Effect of the sulphur atom in the spacial orientation of the 5′-U s^{2}U U-3′ microhelix of B-type optimized at the M062X/6-31G(d,p) level.

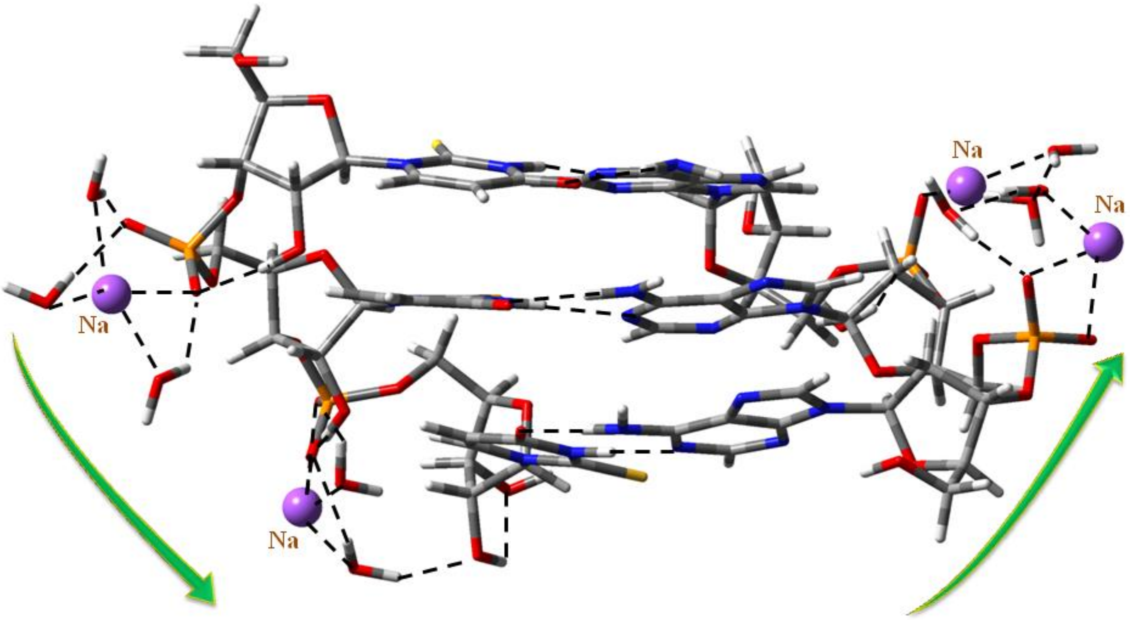

Fig. S13. Effect of the sodium atoms and the water molecules (10 in total) in the spacial orientation of the 5′-s^{2}U s^{2}U s^{2}U-3′ microhelix of B-type optimized at the M062X/6-31G(d,p) level.

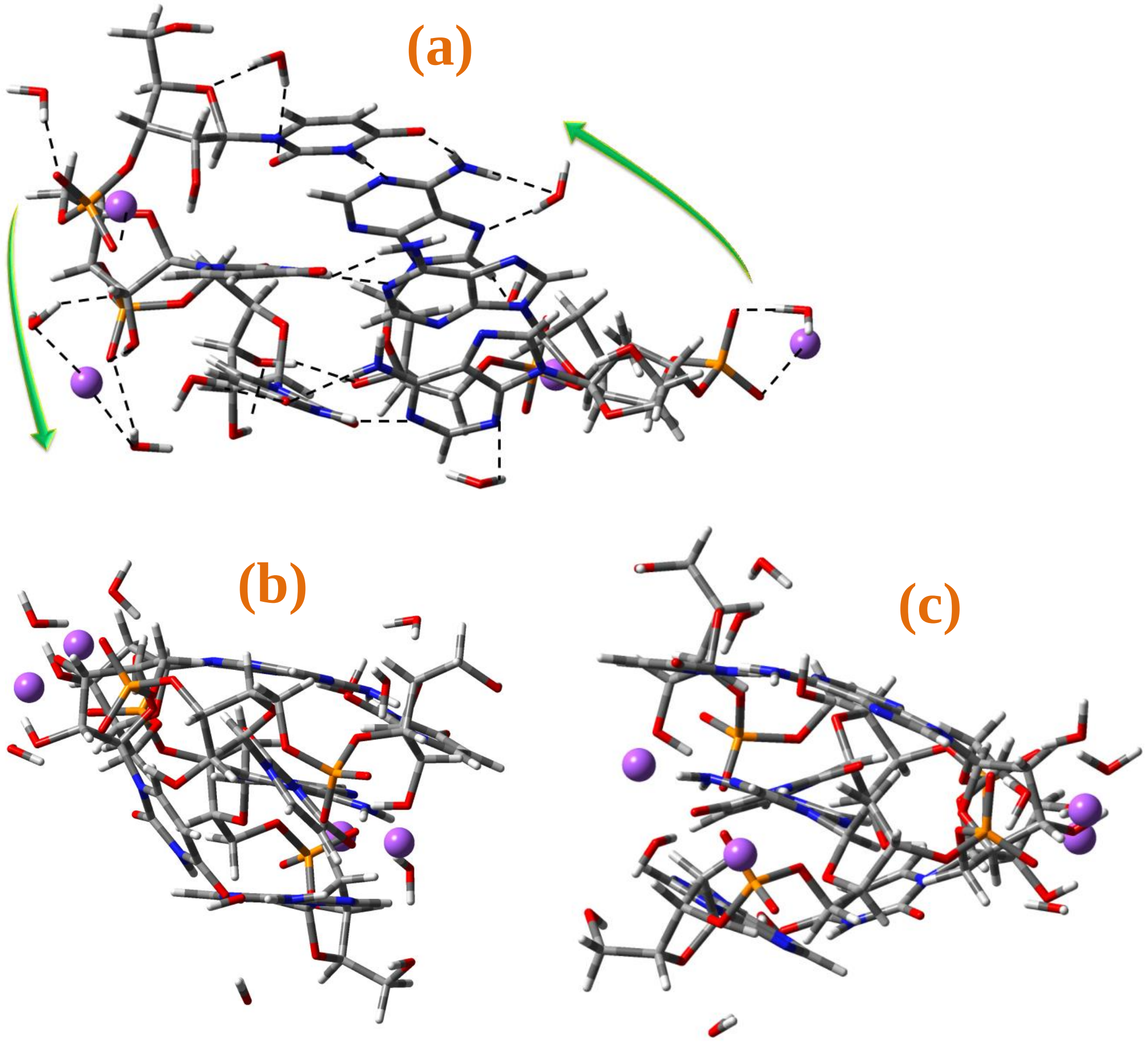

Fig. S14. Three views of the microhelix 5′-UUU-3′ with three nucleotide pairs optimized + 4 Na + 10 H_2O at the B97D/6-31G(d,p) level.

International Journal of *Molecular Sciences*

MDPI

Table S1. Characteristic optimized H-bonds/distances (in Å), bond and torsional angles (in deg.) calculated at M062X/6-31G(d,p) level in the microhelixes with three nucleotide base pairs.

	Structure	(1_0) $H3_0^{U,S}\cdots N1_0^A$	(2_0) $O4_0^{U,S}\cdots H6_0^A$	$N3_0^{U,S}$-H$\cdots N1_0^A$	φ_o^{II}	$N3_{-1}^{U,S}\cdots N3_0^{U,S}/N1_{-1}^A$	$N3_0^{U,S}\cdots N3_1^{U,S}/N1_1^A$	$N1_0^{U,S}\cdots N9_0^A$	$C1'^{U,S}_0\cdots C1'^A_0$	$N1_0^{U,S}$-$C1'^{U,S}_0\cdots C1'^A_0$-$N9_0^A$	$N1_0^{U,S}$-$C1'^{U,S}_0\cdots C1'^A_0$	$C1'^{U,S}_0\cdots C1'^A_0$-$N9_0^A$	$P_1\cdots P_2$	$P_3\cdots P_4$
A-type	**5′-U U U-3′**	1.937	1.923	173.9	-35.7	3.322	3.255	8.879	10.533	-26.8	59.0	51.5	6.396	6.494
	**5′-U U U-3′ (*)**	1.901	1.877	175.9	-24.9	3.300	3.271	9.077	10.918	-25.0	53.6	47.4	6.413	6.854
	**5′-A U A-3′**	1.830	1.971	177.0	7.2	3.246	4.450	8.838	10.376	10.8	60.1	56.8	6.430	6.489
	**5′-A U A-3′ (**)**	1.698	2.065	173.7	5.0	3.193	4.415	8.592	9.898	-3.7	64.3	62.9	6.277	5.505
	**5′-U s^2U U-3′**	1.904	1.919	174.6	-35.3	3.377	3.334	8.864	10.591	-27.1	55.9	51.6	6.349	6.465
	**5′-A s^2U A-3′**	1.925	1.944	178.1	11.6	3.266	4.357	8.956	10.639	12.4	55.0	55.5	6.469	6.571
	**5′-U s^4U U-3′**	2.007	2.324	172.1	-18.7	3.324	3.432	9.032	10.643	-14.3	60.6	52.0	6.426	7.003
	**5′-A s^4U A-3′**	1.942	2.414	170.8	4.8	3.322	4.563	8.802	10.175	5.7	65.2	59.2	6.417	6.475
	**5′-U $s^{2,4}$U U-3′**	2.093	2.408	170.2	-33.2	3.409	3.624	8.908	10.457	-24.6	61.4	54.4	6.389	6.432
	**5′-A $s^{2,4}$U A-3′**	2.141	2.398	173.9	11.3	3.355	4.560	9.066	10.603	11.0	59.7	57.7	6.477	6.574
B-type	**5′-U U U-3′**	1.965	1.815	175.6	-22.8	3.421	3.561	9.146	11.054	-8.9	48.3	49.0	7.213	7.319
	**5′-A U A-3′**	2.009	1.948	174.1	-3.0	3.211	4.752	9.145	10.942	4.0	50.5	52.8	7.058	7.036
	**5′-U s^2U U-3′**	1.964	1.815	175.5	-21.9	3.530	3.657	9.153	11.107	-4.8	45.9	49.2	7.217	7.290
	**5′-A s^2U A-3′**	2.016	1.948	173.3	-10.2	3.251	4.911	9.141	10.961	6.6	49.0	53.2	7.203	7.064
	**5′-s^2U s^2U s^2U-3′**	2.050	1.812	172.6	-21.2	3.552	3.804	9.258	11.247	-0.8	44.8	48.6	7.189	7.291
	**5′-U s^4U U-3′**	1.946	2.311	171.8	-22.6	3.493	3.733	8.974	10.685	-17.0	54.8	52.4	7.216	7.336
	**5′-A s^4U A-3′**	2.072	2.411	175.0	3.2	3.237	4.817	9.031	10.626	1.8	57.2	56.2	6.987	6.959
	**5′-U $s^{2,4}$U U-3′**	2.093	2.290	174.9	-21.7	3.588	3.767	9.177	11.005	-8.8	50.7	51.2	7.252	7.286
	**5′-A $s^{2,4}$U A-3′**	2.212	2.408	177.1	-6.3	3.317	4.998	9.224	10.906	4.6	53.7	53.7	7.050	7.036
	**5′-$s^{2,4}$U $s^{2,4}$U $s^{2,4}$U-3′**	2.105	2.338	176.1	-20.6	3.809	3.953	9.196	10.987	-9.5	51.0	52.7	7.186	7.302

2 (*) With type C in strand *II*. (**) A-type with 4 Na + 8 H_2O. [a] O2/S2$\cdots H6_0^A$. Definition: $\varphi_o^{II} = C4_0^{U,S}$-$N3_0^{U,S}\cdots N1_0^A$-$C6_0^A$,

Table S2. Backbone parameters calculated at the M062X/6-31G(d,p) level in the nucleoside base pair with dA and in the central plane *n* of the microhelixes with 3 nucleotide pairs. Endocyclic and exocyclic[a] torsional angles are in degrees, pseudorotational angle P in degrees, total energy (*E*) in a.u. and dipole moments (μ) in Debye.

	Structure	χ	ζ	α	β	γ	δ	ε	ε´	ν_0	ν_1	ν_2	ν_3	ν_4	P[b]	S[c]	ν^{d}_{max}	*E*	μ
A-type	**5′-U U U-3′**	-155.7	-72.6	-69.8	168.4	65.8	84.0	-160.0	-150.0	6.07	-27.45	37.03	-34.11	18.17	9.3	^{3}E	37.5	-7358.843956	14.347
	**5′-U U U-3′ (*)**	-155.3	-72.1	-70.5	169.0	65.2	84.1	-162.0	-149.9	6.29	-27.74	37.32	-34.25	18.09	9.1	^{3}E	37.8	-7358.846524	17.354
	**5′-A U A-3′**	-163.5	-71.7	-72.4	166.6	58.2	73.6	50.2	-110.4	7.46	-31.62	42.56	-38.86	20.24	8.7	$^{3}_{2}T$	43.1	-7437.450232	6.358
	**5′-A U A-3′ (**)**	-165.9	-73.5	-62.4	160.6	53.4	70.1	50.6	-129.6	6.56	-33.05	45.37	-42.63	22.94	10.5	^{3}E	46.1	-8698.153226	9.223
	**5′-U s²U U-3′**	-155.0	-73.9	-69.1	168.9	65.9	84.8	-157.4	-155.2	3.49	-24.48	34.81	-33.42	19.31	13.0	^{3}E	35.7	-7681.792785	14.104
	**5′-A s²U A-3′**	-163.5	-73.6	-70.7	165.7	58.8	72.9	52.4	-124.0	6.64	-31.29	42.86	-39.52	21.16	9.8	^{3}E	43.5	-7760.396037	7.038
	**5′-U s⁴U U-3′**	-155.6	-71.5	-71.2	169.0	65.3	83.9	-163.2	-147.9	8.19	-29.47	38.29	-34.13	16.84	6.4	$^{3}_{2}T$	38.5	-7681.789089	9.461
	**5′-A s⁴U A-3′**	-165.9	-72.1	-70.8	166.5	57.9	73.7	48.7	-109.8	8.28	-32.49	43.17	-39.01	19.81	7.7	$^{3}_{2}T$	43.6	-7760.400262	6.034
	**5′-U s²,⁴U U-3′**	-154.1	-74.7	-68.7	168.4	65.0	84.9	-161.0	-152.1	4.54	-25.56	35.61	-33.59	18.76	10.6	^{3}E	36.1	-8004.735144	14.676
	**5′-A s²,⁴U A-3′**	-165.0	-74.0	-69.5	165.6	58.1	74.0	51.1	-118.5	6.74	-31.20	42.63	-39.17	20.87	9.6	^{3}E	43.2	-8083.342737	7.294
B-type	**5′-U U U-3′**	-113.3	-131.2	-64.5	166.8	55.2	143.4	-176.9	-16.4	-29.27	43.12	-39.68	24.14	3.09	157.2	^{2}E	43.0	-7358.876542	11.596
	**5′-A U A-3′**	-112.0	-112.4	-60.2	172.0	54.8	137.8	-173.5	-21.7	-35.07	45.14	-37.38	18.41	10.31	147.9	$^{2}_{1}T$	44.1	-7437.469906	11.556
	**5′-U s²U U-3′**	-112.7	-139.7	-65.7	162.2	54.3	144.7	-176.5	-18.0	-30.51	44.40	-40.44	24.46	3.60	156.5	^{2}E	44.1	-7681.820323	13.074
	**5′-A s²U A-3′**	-113.7	-112.6	-61.7	173.1	53.9	137.1	163.4	-5.2	-35.31	47.52	-40.29	21.39	8.48	150.6	$^{2}_{1}T$	46.3	-7760.415733	13.127
	**5′-s²U s²U s²U-3′**	-113.7	-141.7	-66.1	165.1	53.9	146.0	-175.4	-19.9	-28.07	42.81	-40.22	25.63	1.33	159.4	^{2}E	43.0	-8327.706971	15.551
	**5′-U s⁴U U-3′**	-113.0	-128.0	-66.2	170.6	56.3	143.7	-175.9	-17.2	-26.40	40.75	-38.63	24.75	0.86	160.0	^{2}E	41.1	-7681.822975	13.059
	**5′-A s⁴U A-3′**	-110.7	-111.0	-62.3	179.0	54.3	140.5	-171.2	-23.0	-30.06	41.76	-36.98	20.95	5.61	153.6	^{2}E	41.3	-7760.419770	13.338
	**5′-U s²,⁴U U-3′**	-113.3	-144.1	-66.6	160.3	56.0	144.1	-175.7	-18.6	-28.46	42.47	-39.31	24.47	2.30	158.0	^{2}E	42.4	-8004.765034	13.655
	**5′-A s²,⁴U A-3′**	-113.2	-114.6	-63.1	176.1	54.6	141.0	-173.7	-22.1	-31.69	43.62	-38.11	21.41	6.23	152.9	$^{2}_{1}T$	42.8	-8083.362571	12.775
	**5′-s²,⁴U s²,⁴U s²,⁴U -3′**	-115.1	-135.2	-65.2	168.6	55.8	144.7	-174.4	-20.4	-28.29	42.24	-39.16	24.46	2.19	158.2	^{2}E	42.2	-9296.541675	17.574

(*) With type C in strand *II*. (**) A-type with 4 Na + 8 H_2O. [a] χ(O4′-C1′-N1-C2), ζ(C3′$_1$-O3′$_1$-P-O5′), α(O3′$_1$-P-O5′-C5′), β(P-O5′-C5′-C4′), γ(O5′-C5′-C4′-C3′), δ(C5′-C4′-C3′-O3′), ε(C4′-C3′-O3′-P_2), ε´(H2′-O2′-C2′-C3′). [b]When ν_2 is negative, 180° is added to the calculated value of P. [c]Notation used from ref. [68]. [d] $\nu_{max} = \nu_2/\cos P$

Table S3. Several selected parameters[a] calculated at the M062X/6-31G(d,p) level in microhelixes with 3 nucleotide pairs. Distances are in Å and angles in degrees.

	**Structure**	**U_2**	**U_3**	**U_5**	**$M_{-1}Q_{-1}R_{-1}$**	**$M_0Q_0R_0$**	**$M_1Q_1R_1$**	**Dz_{-1}**	**Dz_0**	**d**	**θp**	**R**	**INC**	**aM_0O_0Z**	**cR_0O_0Z**
Nucleotides 3 pairs (A-type)	**5′-U U U-3′**	159.3	167.5	160.7	160.5	167.3	171.8	3.357	3.308	17.178	-30.6	8.603	12.0	52.5	-89.2
	**5′-U U U-3′ (*)**	157.2	167.9	163.9	174.5	178.0	178.2	3.352	3.292	17.717	-26.1	9.160	17.4	-1.9	-16.5
	**5′-A U A-3′**	118.1	155.7	142.0	172.2	169.3	164.8	3.249	3.822	17.386	2.5	8.972	8.1	8.6	6.2
	**5′-A U A-3′ (**)**	117.9	155.0	136.2	173.5	166.6	169.3	3.220	3.584	17.009	-6.2	8.867	10.2	6.5	9.8
	**5′-U s^2U U-3′**	159.7	165.5	160.0	162.8	168.8	172.5	3.433	3.369	17.232	-30.8	8.625	12.2	63.7	82.2
	**5′-A s^2U A-3′**	118.0	156.1	143.2	172.8	170.9	163.9	3.274	3.713	17.568	4.4	8.996	8.3	16.6	7.7
	**5′-U s^4U U-3′**	150.2	165.2	167.2	170.5	172.3	176.2	3.561	3.225	17.139	-20.7	9.466	17.8	11.4	-18.0
	**5′-A s^4U A-3′**	113.5	148.0	142.5	170.3	166.8	165.2	3.262	3.609	17.260	-3.2	9.042	8.4	4.4	10.6
	**5′-U $s^{2,4}$U U-3′**	154.5	171.7	168.4	159.0	165.4	171.3	3.585	3.481	17.243	-30.6	8.654	12.0	42.2	-74.4
	**5′-A $s^{2,4}$U A-3′**	113.1	148.4	144.5	169.9	170.4	164.3	3.304	3.590	17.626	3.1	9.106	7.8	17.4	4.8
Nucleotides 3 pairs (B-type)	**5′-U U U-3′**	147.0	162.8	167.2	172.8	175.4	170.7	3.022	2.880	16.594	-21.8	9.916	15.7	24.8	-31.7
	**5′-A U A-3′**	110.4	152.7	141.0	178.0	173.5	172.3	3.085	3.367	17.121	-5.6	11.001	10.2	6.7	1.0
	**5′-U s^2U U-3′**	144.9	158.7	168.7	170.9	176.7	174.3	2.988	3.200	16.628	-20.6	9.958	16.0	23.7	-27.7
	**5′-A s^2U A-3′**	115.5	156.5	144.0	176.3	173.1	171.7	3.153	3.589	16.784	-11.2	11.182	7.6	1.1	2.7
	**5′-s^2U s^2U s^2U-3′**	144.8	160.2	170.6	169.8	176.8	174.6	2.849	3.244	16.794	-17.7	11.577	16.6	28.1	-25.0
	**5′-U s^4U U-3′**	144.5	164.3	173.0	170.4	172.9	168.4	3.339	2.973	16.221	-26.8	10.227	18.1	24.4	-38.7
	**5′-A s^4U A-3′**	106.9	147.0	139.4	176.1	170.5	171.0	3.108	3.002	16.849	-3.0	10.998	12.5	14.2	-2.0
	**5′-U $s^{2,4}$U U-3′**	141.8	159.7	173.8	170.5	175.1	173.1	3.282	3.104	16.509	-23.3	10.048	17.3	25.2	-33.0
	**5′-A $s^{2,4}$U A-3′**	110.4	151.1	144.3	178.3	172.8	172.0	3.198	3.203	16.952	-8.6	11.199	11.9	11.3	-2.6
	**5′-$s^{2,4}$U $s^{2,4}$U $s^{2,4}$U -3′**	148.9	164.5	172.6	164.1	175.2	168.6	3.082	3.174	16.545	-21.9	10.447	17.5	26.8	-33.3

(*) With type C in strand *II*. (**) A-type with 4 Na + 8 H_2O. [a] Notation used according to ref. [28]: $U_2 \equiv P_{-1}P_0P_1$, $U_3 \equiv Q_{-1}Q_0Q_1$, $U_5 \equiv R_{-1}R_0R_1$, $Dz_{-1} \equiv bM_0$, $Dz_o \equiv aM_0$, d (microhelix diameter) = mq, θp (propeller twist) $\equiv U_1U_4$ (aM_0R_0c), R (helical radius) $\equiv mO_o$, INC (inclination) $\equiv qO_oC8_o$.

Table S4. Several selected intermolecular/intramolecular H-bonds (in Å) found in the optimized microhelixes with 3 nucleotide pairs at the M062X/6-31G(d,p) level. The microhelixes with Na atoms and water molecules appear with neutral charge.

Structure		①$_{-1}$ $H3^{U,S}_{-1}\cdots N1^{A}_{-1}$	①$_{1}$ $H3^{U,S}_{1}\cdots N1^{A}_{1}$	②$_{-1}$ $O4^{U,S}_{-1}$ $4'^{U,S}$… $H6^{A-1}_{-1}$	②$_{1}$ $O4^{U,S}_{1}\cdots H6^{A}_{1}$	④$_{-1}$ $H2'^{U,S}_{-1}\cdots O4'^{U,S}_{0}$	④$_{0}$ $H2'^{U,S}_{0}\cdots O4'^{U,S}_{1}$	⑧$_{-1}$ $H2'^{U,S}_{-1}\cdots O7'^{U,S}_{-1}$	⑧$_{0}$ $H2'^{U,S}_{0}\cdots O7'^{U,S}_{0}$
A-type	**5′-U U U-3′**	1.748	1.819	2.107	1.900	1.886	2.075	-	-
	**5′-U U U-3′ (*)**	1.718	1.906	2.106	1.865	1.885	2.063		
	**5′-A U A-3′**	1.784	1.766	1.927	2.063	1.936	1.959	-	-
	**5′-A U A-3′ (**)**	1.785	1.797	1.879	1.957	2.050	1.938		
	**5′-U s²U U-3′**	1.729	1.805	2.138	1.896	1.884	2.192	-	-
	**5′-A s²U A-3′**	1.839	1.782	1.886	2.043	1.938	1.945		
	**5′-U s⁴U U-3′**	1.709	1.828	2.216	1.902	1.901	2.014	-	-
	**5′-A s⁴U A-3′**	1.763	1.756	1.965	2.083	1.974	1.954		
	**5′-U s²,⁴U U-3′**	1.738	1.818	2.127	1.903	1.881	2.096	-	-
	**5′-A s²,⁴U A-3′**	1.813	1.777	1.916	2.060	1.967	1.938		
B-type	**5′-U U U-3′**	1.716	1.866	2.086	1.877	-	-	1.556	1.627
	**5′-A U A-3′**	1.854	1.699	1.886	2.005	-	-	1.569	1.647
	**5′-U s²U U-3′**	1.704	1.877	2.146	1.853	-	-	1.555	1.633
	**5′-A s²U A-3′**	1.906	1.695	1.854	2.013	-	-	1.576	1.602
	**5′-s²U s²U s²U-3′**	1.777	1.936	2.082	1.831	-	-	1.531	1.638
	**5′-U s⁴U U-3′**	1.720	1.838	2.188	1.918	-	-	1.553	1.617
	**5′-A s⁴U A-3′**	1.811	1.680	1.914	2.057	-	-	1.563	1.649
	**5′-U s²,⁴U U-3′**	1.713	1.862	2.179	1.891	-	-	1.551	1.624
	**5′-A s²,⁴U A-3′**	1.870	1.697	1.889	2.018	-	-	1.560	1.653
	**5′-s²,⁴Us²,⁴Us²,⁴U -3′**	2.026	2.073	2.585	2.396	-	-	1.525	1.616
+ 4 Na	**5′-U U U-3′**	1.722	1.788	2.243	1.892			1.780	1.804
	**5′-s²U s²U s²U-3′**	1.811	1.872	2.132	1.829			1.775	1.843
	**5′-s⁴U s⁴U s⁴U-3′**	1.845	1.858	> 2.5	2.373			1.784	1.803
+ 4 Na + 20 H_2O	**5′-U U U-3′**	1.769	1.734	2.233	1.979			1.668	1.625
	**5′-s²U s²U s²U-3′**	1.866	1.817	2.149	1.842			1.699	1.582
	**5′-s⁴U s⁴U s⁴U-3′**	1.939	1.818	2.518	2.488			1.697	1.634
	**5′-s²,⁴Us²,⁴U s²,⁴U-3′**	2.120	2.016	2.485	2.353			1.659	1.596

(*) With type C in strand *II*. (**) A-type with 4 Na + 8 H_2O. [a] $N3^{C}_{-1}\cdots H1^{G}_{-1}$- $N1^{G}_{-1}$ [b] $N3^{C}_{1}\cdots H1^{G}_{1}$- $N1^{G}_{1}$ [c] $N4^{C}_{-1}$-$H4^{C}_{-1}\cdots O6^{G}_{-1}$ [d] $N4^{C}_{1}$-$H4^{C}_{1}\cdots O6^{G}_{1}$
